# The influence of body position on bioelectrical impedance spectroscopy measurements in young children

**DOI:** 10.1038/s41598-021-89568-8

**Published:** 2021-05-14

**Authors:** Jaz Lyons-Reid, Leigh C. Ward, Mya-Thway Tint, Timothy Kenealy, Keith M. Godfrey, Shiao-Yng Chan, Wayne S. Cutfield

**Affiliations:** 1 Liggins Institute, University of Auckland, Auckland, New Zealand; 2 School of Chemistry and Molecular Biosciences, The University of Queensland, Brisbane, Australia; 3 Singapore Institute for Clinical Sciences, Agency for Science, Technology and Research (A*STAR), Singapore, Singapore; 4 Department of Obstetrics and Gynaecology, Yong Loo Lin School of Medicine, National University of Singapore, Singapore, Singapore; 5 Department of Medicine and Department of General Practice and Primary Health Care, University of Auckland, Auckland, New Zealand; 6 MRC Lifecourse Epidemiology Unit, University of Southampton, Southampton, UK; 7 NIHR Southampton Biomedical Research Centre, University of Southampton and University Hospital Southampton NHS Foundation Trust, Southampton, UK; 8 A Better Start – National Science Challenge, University of Auckland, Auckland, New Zealand

**Keywords:** Paediatrics, Paediatric research

## Abstract

Bioelectrical impedance techniques are easy to use and portable tools for assessing body composition. While measurements vary according to standing vs supine position in adults, and fasting and bladder voiding have been proposed as additional important influences, these have not been assessed in young children. Therefore, the influence of position, fasting, and voiding on bioimpedance measurements was examined in children. Bioimpedance measurements (ImpediMed SFB7) were made in 50 children (3.38 years). Measurements were made when supine and twice when standing (immediately on standing and after four minutes). Impedance and body composition were compared between positions, and the effect of fasting and voiding was assessed. Impedance varied between positions, but body composition parameters other than fat mass (total body water, intra- and extra-cellular water, fat-free mass) differed by less than 5%. There were no differences according to time of last meal or void. Equations were developed to allow standing measurements of fat mass to be combined with supine measurements. In early childhood, it can be difficult to meet requirements for fasting, voiding, and lying supine prior to measurement. This study provides evidence to enable standing and supine bioimpedance measurements to be combined in cohorts of young children.

## Introduction

Bioelectrical impedance techniques allow quick, easy measurement of body composition including, total body water (TBW), fat mass (FM), and fat-free mass (FFM). Multi-frequency techniques, including multi-frequency bioimpedance analysis (MFBIA) and bioimpedance spectroscopy (BIS), are further able to distinguish between intracellular (ICW) and extracellular fluids (ECW)^1,2^. Although not widely used in early childhood, bioimpedance techniques are easy to administer, are inexpensive, and require less co-operation from the child due to their fast measurement time in comparison to other widely used methods, such as dual-energy X-ray absorptiometry (DXA). However, there are many factors that may influence bioimpedance measurements and thus require standardisation^3^. These factors may be amplified in infants and young children, where compliance is a particular challenge^4^. One such factor is the requirement for children to lie supine for extended periods prior to measurement.

Brantlov et al.^5^ reported that of 71 studies identified which used bioelectrical impedance analysis to estimate body composition in populations of healthy children, authors did not consistently report in what body position (i.e., standing or supine) measurements were obtained. Of concern, only 21% reported how long the child was in the position prior to measurement. In adults, it has been shown that standing and supine measurements are not interchangeable and that it takes approximately 5 min for fluid stabilisation to occur to allow measurement of TBW^6^ and extended periods to establish ECW and ICW stabilisation^6,7^, which correspond to changes in impedance values^8^. As such, adult guidelines recommend that bioimpedance measurements be made in the supine position after 4 to 10 min have elapsed^9,10^. However, no guidelines exist for paediatric populations.

While studies in young children have explored some of the factors which influence impedance measurements, such as movement^11^ and electrode placement^11–13^, no study has evaluated the effect of body position. In young children, it may be more feasible to obtain bioimpedance measurements while the child is standing; however, it is unclear whether measurements taken in alternate body positions are interchangeable. In addition to recommendations about body position, adult guidelines state that bioimpedance measurements should be made when the subject is fasted and has voided their bladder^9,10^, as impedance parameters have been found to be modestly reduced following a meal or bladder void^8,14,15^; however, it is unclear what effect, if any, these factors may have on measurements in young children. Therefore, the aim of this study was to determine whether BIS measurements obtained in different body positions can be used interchangeably and whether fasting and bladder voiding influence associations.

## Methods

### Subjects

A convenience sample of children aged 3.38 years was selected from the Auckland site of the Nutritional Intervention Preconception and During Pregnancy to Maintain Healthy Glucose Metabolism and Offspring Health (“NiPPeR”) study^16^. Data were obtained from 50 children selected based on compliance with the NiPPeR BIS protocol (i.e., the child laid supine for ≥ 4 min prior to the initial measurement).

### Ethics

The NiPPeR trial was registered on 16 July 2015 with ClinicalTrials.gov (NCT02509988, Universal Trial Number U1111-1171-8056); ethics approval was granted by the Northern A Health and Disability Ethics Committee (15/NTA/21/AM20). Written informed consent was obtained from the parents/guardians of the study subjects. All procedures in this study were conducted according to the ethical principles and guidelines laid down in the Declaration of Helsinki.

### Experimental design

Children were measured in three body positions. First, as per the recommendation of Brantlov et al.^3^ (based on adult guidelines^9,10^), children were measured supine on non-conductive examination tables with the legs separated and arms by their sides without skin-to-skin contact between arms and the trunk, after at least four minutes had elapsed (thus allowing fluid stabilisation). Second, the children were measured immediately (within one minute) on standing (from being supine) while maintaining correct abduction of the arms and legs. Finally, children were measured in the same standing position after at least four minutes had elapsed. During this period, children were required to remain upright (standing or seated). For each body position, electrode placement remained the same, and it was ensured that the leads were not tangled or touching any metal surfaces. It was not possible to ensure that the leads were not touching the ground during the standing measurements due to the placement of the electrodes.

In addition, whether the child had fasted or voided their bladder was recorded. The effect of consumption of food or drink on impedance measurements has not been explored in preschool aged children. Evidence from infancy suggests that it is time after consumption, rather than volume, that is important^11^. Thus, time of last meal or drink (> 2 h ago, 1–2 h ago, 30 min–1 h ago, or ≤ 30 min ago) was recorded, as was time of last void. Time of last void was categorised according to whether or not the child had voided their bladder within half an hour of measurement. If the child consumed any food or fluid, or voided their bladder between measurement positions, this was recorded. These children were excluded from analyses evaluating the effect of fasting and voiding on differences in impedance between body positions (n = 5).

### Bioelectrical impedance spectroscopy

Bioimpedance measurements were made with the ImpediMed SFB7 device (ImpediMed, Brisbane, Australia). This device measures bioimpedance parameters over a frequency range of 3 to 1000 kHz, resulting in 256 measurements per assessment^17^. Instrument calibration was checked daily prior to use using a test cell provided by the manufacturer. ImpediMed single-tab gel electrodes (25 $\times$ 23 mm) were used to attach sense leads to the left or right dorsum wrist and ankle, and the source leads to the palm at the metacarpal heads and the sole at the metatarsal heads on the same side of the body^18^. No differences in impedance parameters were observed between measurement sides (all *p* > 0.05). Prior to careful application of the electrodes, the skin was cleaned with 70% isopropyl alcohol wipes and allowed to dry. Any clothing with metal (e.g., clips or buckles) was removed prior to measurement to avoid electrical interference. Otherwise, clothing was only removed to access electrode sites. For each body position, measurements were made in triplicate using the “continuous” setting of the device; each measurement taking less than a second. Cole plots were examined to ensure data quality and measurements were repeated if movement occurred.

Data was analysed using BioImp software version 5.4.0.3 (ImpediMed), using the default settings [frequency range 5–500 kHz, automatic time delay (Td) correction on, no data rejection limit]. The impedance values of interest were as follows: resistance at 0 kHz, R_0_; resistance at infinite kHz, R_∞_; impedance at 50 kHz, Z_50_; resistance at 50 kHz, R_50_; reactance at 50 kHz, Xc_50_; and impedance at the characteristic frequency, Z_c_.

### Assessment of body composition

Standing height was measured in triplicate to the nearest 0.1 cm using a calibrated SECA 213 portable stadiometer (SECA, Hamburg, Germany), with median height being used in analyses, while a single weight measurement was obtained to the nearest 100 g using calibrated SECA 899 scales. Along with sex, these values were used to compute body composition measures using two methods: mixture theory in combination with Cole modelling [i.e., the SFB7’s default equations and constants: resistivity of ECW (ρECW) and ICW (ρICW)—females 235.5 and 894.2 Ω/cm, and males 273.9 and 937.2 Ω/cm, respectively; body density (Db) 1.05 g/L; body proportion factor (Kb) 4.30; and hydration factor (HF) 0.732]^17,19,20^, and an empirically-derived regression equation^21^.

The SFB7 provides the following body composition values: TBW, ECW, ICW, FFM, and FM. However, the coefficients used by the SFB7 may not be suitable for use in small children;^22,23^ therefore, measured resistance at 50 kHz was used in a previously published empirically derived regression equation for FFM (FFM_Rush_), which was developed using DXA among a cohort of New Zealand 2-year-olds^21^. The reported equation is as follows:

$${\text{FFM}}_{{{\text{Rush}}}} { }\left( {{\text{kg}}} \right) = 0.367\frac{{{\text{height }}\left( {{\text{cm}}} \right)^{2} }}{{{\text{resistance}}}} + 0.188 {\text{weight }}\left( {{\text{kg}}} \right) + 0.077{\text{ height }}\left( {{\text{cm}}} \right) + 0.273{\text{ sex }}\left( {{\text{male}} = 1,{\text{ female}} = 0} \right)$$

FM (FM_Rush_) was computed from FFM considering a two-compartment model of body composition^24^ and the following equation:

$${\text{FM}}_{{{\text{Rush}}}} { }\left( {{\text{kg}}} \right) = {\text{Weight }}\left( {{\text{kg}}} \right) - {\text{FFM}}_{{{\text{Rush}}}} { }\left( {{\text{kg}}} \right)$$

### Statistical methods

Mean (SD) bioimpedance parameters (R_∞_, R_0_, Z_c_, R_50_, Z_50_, and Xc_50_) and body composition values (TBW_SFB7_, ECW_SFB7_, ICW_SFB7_, FFM_SFB7_, FM_SFB7_, FFM_Rush_, and FM_Rush_) were assessed in each of the body positions (supine, standing ≤ 1 min, and standing ≥ 4 min), with sex differences in impedance parameters being explored using independent samples *t* tests. Differences in impedance and body composition between supine and both standing positions were assessed using repeated measures ANOVA with Bonferroni post hoc testing, with differences in body composition values between supine and standing (≥ 4 min) positions being presented as percentage differences. The effect of fasting and bladder voiding on differences in impedance measurements was assessed using one-way ANOVA and independent samples *t* tests.

In order to develop equations to allow adjustment of bioimpedance parameters obtained while standing, thus allowing their use in equations where supine body position is indicated, the cohort was split into development (70%) and validation cohorts (30%) using a random number generator within SPSS version 26 (IBM Corp, Armonk, NY, USA). Among the development cohort (n = 35), for each impedance parameter, simple linear regression was used to develop an equation to adjust impedance values obtained while standing (≥ 4 min) to be comparable to those obtained while supine. These resulting equations were then applied to the validation cohort (n = 15). The equations were also applied to standing (≤ 1 min) measurements among the validation cohort to further elucidate the importance of time spent standing. Impedance parameters from supine measurements were compared to the adjusted standing measurements using paired samples *t* tests and Bland–Altman’s methods^25^. All tests were two-tailed and were performed within SPSS. *P* values ≤ 0.05 were considered statistically significant.

## Results

### Demographics

The sample comprised 50 children, 20 of whom were male and 30 female. On average, the children were 3.38 years old, with boys being somewhat taller and heavier than girls (Table 1).

**Table 1:** Study population characteristics.

	Mean (SD) population characteristics		
	Boys (n = 20)	Girls (n = 30)	All (n = 50)
Age (years)	3.38 (0.14)	3.38 (0.15)	3.38 (0.14)
Height (cm)	99.26 (3.63)	98.96 (3.87)	99.08 (3.74)
Weight (kg)	16.08 (1.72)	15.74 (2.04)	15.88 (1.91)
BMI_SDS_	0.60 (0.19)	0.45 (0.15)	0.51 (0.83)

### Sex effects

Mean impedance parameters were larger among girls than boys in each of the body positions. These differences were significant, with the exception of standing (≥ 4 min) mean reactance at 50 kHz (*p* = 0.065). In contrast, the means of the differences in impedance parameters between supine and standing (≥ 4 min) positions were not significantly different between sexes, with the exception of reactance at 50 kHz (*p* < 0.001). Given the similarity in all other mean differences, further comparisons were made using the entire cohort.

### Differences between standing and supine

Mean impedance parameters for supine and standing (< 1 min and ≥ 4 min) measurements are presented in Table 2. There were significant differences between body positions in all impedance parameters (p < 0.001). Post-hoc comparisons revealed that there were differences between impedance parameters obtained when supine compared to both standing positions (all p < 0.001), with supine values larger than those obtained when standing. Impedance parameters were generally higher when obtained standing immediately from supine (< 1 min) compared to standing (≥ 4 min), with the exception of reactance at 50 kHz where the reverse was true, but these differences were not statistically significant. There were also significant differences (*p* < 0.001) in all body composition parameters between supine and both standing positions (Table 3). However, these differences were probably of little clinical significance, with percentage differences of less than five percent, with the exception of FM, which exhibited both greater percentage differences and greater variability.

**Table 2:** Mean bioimpedance parameters when the participants were measured supine and standing (< 1 min and ≥ 4 min).

	Mean (SD) impedance parameters		
	Supine	Standing (< 1 min)	Standing (≥ 4 min)
R_0_ (Ω)	813.5 (76.6)	789.3 (76.7)	786.1 (77.1)
R_∞_ (Ω)	598.3 (63.8)	578.9 (65.0)	576.2 (67.1)
Z_c_ (Ω)	709.0 (69.7)	687.1 (70.3)	684.2 (71.5)
Z_50_ (Ω)	746.3 (72.5)	724.6 (72.5)	720.9 (73.1)
R_50_ (Ω)	743.8 (72.5)	722.2 (72.5)	718.5 (73.1)
Xc_50_ (Ω)	60.1 (7.2)	57.9 (6.8)	58.1 (6.6)

*R*_*0*_ resistance at 0 kHz, *R*_*∞*_ resistance at infinite kHz, *Z*_*c*_ impedance at the characteristic frequency, *Z*_*50*_ impedance at 50 kHz, *R*_*50*_ resistance at 50 kHz, *Xc*_*50*_ reactance at 50 kHz.

**Table 3:** Mean body composition values when the participants were measured supine and standing (< 1 min and ≥ 4 min).

	Mean (SD) body composition values			% difference^a^
	Supine	Standing (< 1 min)	Standing (≥ 4 min)	
TBW_SFB7_ (L)	9.14 (1.28)	9.36 (1.31)	9.40 (1.35)	− 2.73 (0.285)
ECW_SFB7_ (L)	4.08 (0.56)	4.17 (0.58)	4.18 (0.58)	− 2.31 (1.49)
ICW_SFB7_ (L)	5.06 (0.79)	5.19 (0.81)	5.22 (0.82)	− 3.14 (4.71)
FFM_SFB7_ (kg)	12.49 (1.75)	12.79 (1.79)	12.84 (1.84)	− 2.73 (2.85)
FM_SFB7_ (kg)	3.38 (0.92)	3.09 (0.94)	3.03 (1.04)	13.75 (22.01)
FFM_Rush_ (kg)	15.63 (1.29)	15.78 (1.30)	14.81 (1.32)	− 1.12 (0.76)
FM_Rush_ (kg)	0.25 (0.94)	0.10 (0.92)	0.07 (0.93)	9.12 (140.60)

^a^Percentage difference between mean supine and standing (≥ 4 min) body composition values.

*TBW*_*SFB7*_ total body water from ImpediMed SFB7 built-in equation, *ECW*_*SFB7*_ extracellular water from ImpediMed SFB7 built-in equation, *ICW*_*SFB7*_ intracellular water from ImpediMed SFB7 built-in equation, *FFM*_*SFB7*_ fat-free mass from ImpediMed SFB7 built-in equation, *FM*_*SFB7*_ fat mass from ImpediMed SFB7 built-in equation, *FFM*_*Rush*_ fat-free mass from Rush et al. 2013 equation, *FM*_*Rush*_ fat mass from Rush et al. ^21^ equation.

### Effect of fasting and voiding

Among children who did not eat, drink, or void between measurements (n = 45), there was no clear pattern (i.e., increasing or decreasing across categories) in mean impedance values according to category of last meal (< 30 min ago, 30 min–1 h ago, 1–2 h ago, or > 2 h ago). Furthermore, differences in impedance between standing (≥ 4 min) and supine measurements (i.e., mean differences) did not vary significantly according to category of last meal (*p* values: R_0_ = 0.94, R_∞_ = 0.30, Z_c_ = 0.64, Z_50_ = 0.80, R_50_ = 0.79, Xc_50_ = 0.59). However, most of the children consumed food within half an hour of measurement. Therefore, the groups 30 min to 1 h (n = 7), 1 to 2 h (n = 9), and over 2 h (n = 5) were collapsed, and differences were assessed using an independent samples *t* test. Although there was a trend for greater impedance and resistance, but reduced reactance among those who had not eaten within half an hour of measurement, there remained no significant differences in mean impedance parameters (all *p* > 0.10), or in mean differences in impedance parameters between supine and standing (≥ 4 min) positions (*p* values: R_0_ = 0.70, R_∞_ = 0.86, Z_c_ = 0.74, Z_50_ = 0.58, R_50_ = 0.58, Xc_50_ = 0.83).

Mean impedance parameters were not statistically different between children who had not voided within half an hour of measurement compared to those who had. Likewise, there were no significant variations in the mean differences of impedance parameters according to whether or not the child had voided (*p* values: R_0_ = 0.55, R_∞_ = 0.16, Z_c_ = 0.71, Z_50_ = 0.84, R_50_ = 0.86,). Although, there was a borderline significant difference in reactance at 50 kHz, with mean differences being higher among those who had not voided, compared to those who had (*p* = 0.062).

### Adjustment equations

As there were statistically significant differences between supine and standing positions, equations were developed to allow impedance measurements obtained when standing to be adjusted to be comparable to those obtained while supine (Table 4). The development cohort (n = 35) was not different from the validation cohort (n = 15) in age, sex, height, weight, or BMI *z* score (all *p* > 0.05).

**Table 4:** Regression equations developed in development sub-group (n = 35) to allow measurements obtained when standing (≥ 4 min) to be comparable to those obtained when supine.

	Equation	R	R^2^
R_0_^supine^	31.138 + 0.996 R_0_^standing^	0.977	0.954
R_∞_^supine^	39.498 + 0.970 R_∞_^standing^	0.972	0.945
Z_c_^supine^	30.659 + 0.992 Z_c_^standing^	0.980	0.960
Z_50_^supine^	21.978 + 1.005 Z_50_^standing^	0.979	0.959
R_50_^supine^	21.720 + 1.005 R_50_^standing^	0.980	0.960
Xc_50_^supine^	3.986 + 0.967 Xc_50_^standing^	0.925	0.856

*R*_*0*_ resistance at 0 kHz, *R*_*∞*_ resistance at infinite kHz, *Z*_*c*_ impedance at the characteristic frequency, *Z*_*50*_ impedance at 50 kHz, *R*_*50*_ resistance at 50 kHz, *Xc*_*50*_ reactance at 50 kHz.

When the adjustment equations were applied to the validation cohort, there were no significant differences in mean impedance values between supine and adjusted standing measurements (all *p* > 0.05). Bland–Altman analysis revealed small biases and narrow limits of agreement. These are expressed as absolute values and as percentages of mean supine impedance values (Table 5). The equations were subsequently applied to standing (≤ 1 min) measurements, and there were no significant differences between the adjusted and supine values (all *p* > 0.05). Bias was larger but was still less than 1% of mean supine impedance; however, limits of agreement were marginally narrower (supplementary Table 1).

**Table 5:** Bioimpedance body position adjustment equations applied to standing (≥ 4 min) measurements in validation sub-group (n = 15).

	Validation cohort (n = 15)		T test		Bland–Altman		
	Mean	SD	t	*p*	Bias	Limits of agreement	
						Lower	Upper
**R**_**0**_							
Supine	798.59	73.21	− 0.369	0.718	− 1.82	− 39.33	35.69
Standing (adjusted)	800.41	78.70			− 0.23%	− 4.92%	4.47%
**R**_**∞**_							
Supine	581.27	62.84	− 0.170	0.867	− 0.93	− 42.50	40.64
Standing (adjusted)	582.20	71.66			− 0.16%	− 7.31%	6.99%
**Z**_**c**_							
Supine	693.22	67.64	− 0.260	0.799	− 1.22	− 36.89	34.45
Standing (adjusted)	694.44	75.52			− 0.18%	− 5.32%	4.97%
**Z**_**50**_							
Supine	730.40	71.46	− 0.017	0.987	− 0.08	− 36.57	36.41
Standing (adjusted)	730.49	78.16			− 0.01%	− 5.01%	4.98%
**R**_**50**_							
Supine	727.82	71.56	0.026	0.980	0.13	− 36.46	36.71
Standing (adjusted)	727.70	78.30			0.02%	− 5.01%	5.04%
**Xc**_**50**_							
Supine	61.01	5.57	− 0.264	0.795	− 0.26	− 7.82	7.29
Standing (adjusted)	61.27	3.38			− 0.43%	− 12.82%	11.95%

*R*_*0*_ resistance at 0 kHz, *R*_*∞*_ resistance at infinite kHz, *Z*_*c*_ impedance at the characteristic frequency, *Z*_*50*_ impedance at 50 kHz, *R*_*50*_ resistance at 50 kHz, *Xc*_*50*_ reactance at 50 kHz.

## Discussion

Although adult guidelines dictate that BIA measurements be made supine after at least 4 min have elapsed^9,10^, it is not always feasible in infants and young children. In our study of 50 young children, impedance measurements differed between body positions, with higher derived TBW, ECW, ICW, and FFM, and lower FM in the standing body position; most of the body composition values differed by less than 5%, with the exception of FM (FM_SFB7_ 13.75% lower and FM_Rush_ 9.12% lower).

A recent study evaluated the effect of body position on phase angle in a cohort of 1298 Mexican children and adolescents aged 4 to 20 years^26^. Phase angle was higher when measured supine than standing, with differences between body positions increasing with increased phase angle, age, and height. However, the children were measured with two different BIA devices, which had different electrode types (metal and adhesive), and thus are not directly comparable.

Another study examined differences in body fluid according to measurement position (standing and supine) in a cohort of 23 boys (6–14 years) and 26 men (23–82 years)^27^. Significant impedance differences were also observed (at 50 and 100 kHz in boys and at 100 kHz in men). No significant differences were seen in TBW, FFM, FM, or percentage of body fat (%BF), but body water shifted so that ECW increased and ICW decreased when standing. This is in contrast to our study, where differences were observed in all body composition values, and both ECW and ICW increased when standing. In adults, Gibson et al.^6^ found that ECW decreased and ICW increased while supine. When standing, although ECW increased incrementally, decreases in ICW were not significant. It has been suggested that it takes extended periods to achieve fluid stabilisation^6,28^, which may explain this observed discrepancy.

We were unable to explore time-course changes in impedance values; however, previous research has suggested that changes in impedance are greatest immediately on recumbence/ standing, and changes thereafter are gradual^8,29^. Furthermore, we observed no significant differences in impedance values when measured immediately on standing, compared to after at least four minutes had elapsed.

Regression equations were developed to allow adjustment of standing BIA measurements to be comparable to measurements obtained while supine, irrespective of the amount of time spent standing (Tables 5 and S1). Previously, regression equations have been developed among adults to allow measurements made while sitting upright in a wheelchair to be comparable to measurements made while supine^30^. Similarly, Rush et al.^31^ developed adjustment factors to convert standing measurement to equate supine in children and adults (categorised: 5–14 years, 15–30 years, 31–59 years, and 60 + years). Our equations may be of benefit in studies in young children that wish to use a previously published prediction equation where supine body position is dictated, but where this may not be achievable.

To our knowledge, this study is the first to explore the influence of body position on bioimpedance measurements in young children (< 5 years). At this age, children are often non-compliant, and it is not feasible to obtain BIA measurements after extended periods of lying. It may be of benefit to take measurements while the child is in an alternative body position, for example, while standing. A limitation of our study was that electrode placement meant that the leads were touching the ground. Although, only the external insulating plastic sheath was in contact, and the leads are actively shielded against electrical interference. Furthermore, the placement used was necessary to maintain adequate separation of the electrodes^9,10^. This methodology meant that use of two different BIA devices was avoided. Although some studies have evaluated the effect of body position using different BIA devices^26,32^, ample evidence suggests that BIA device types are not interchangeable^33–35^. Jensen et al.^26^ used two differing BIA devices in their study and concluded that electrode type explained approximately half of the observed differences in phase angle between body positions when they conducted additional analyses in a cohort of adults. However, this is likely related to the differing electrode positions, in addition to the electrode type (metal vs adhesive).

The effect of fasting has not previously been evaluated in preschool aged children; however, evidence from infancy suggests that impedance parameters do not change significantly when measured pre- and post-feed^11,36^. Although, Sesmero et al.^11^ did observe a general trend for increasing R_0_ with increasing time after feed, but this was only significant among their 1-week-old infants. The effect of bladder voiding has not been evaluated in any paediatric population. In adults, bladder voiding has been associated with a small measurement error of 1.0%^14^. In this study, time of last meal or bladder void were often estimated; however, there were no significant differences in impedance between body positions according to fasting or voiding. Nonetheless, half an hour may not be a sufficient difference in time to evaluate the effect of fasting and voiding. However, it would be not be feasible nor ethical to request young children to refrain from eating or voiding for extended periods to evaluate this further, though a larger study group may provide more clarity on this issue.

Other limitations of this research include that the equations used to estimate body composition might not be appropriate for this cohort, as evidenced by the wide standard deviations for FM. However, the aim of the study was not to accurately estimate body composition; rather, body composition values were used to ascertain if clinically significant differences were apparent between body positions. Nonetheless, we used two different methods for estimating body composition (Rush et al.^21^ and SFB7 equations), and the resulting percentage differences between body positions were comparable. In addition, we did not randomise the order of measurements as inclusion into this sub-study was based on compliance with the NiPPeR protocol. Studies in adults have suggested that position order is not important^6,31,37^. For example, among children and adults assessed both standing prior to lying supine and standing following a supine measurement, the second standing measurement was lower than the first by only approximately 1 ohm^31^. Furthermore, in our cohort, we did not standardise measurement side and we were not able to assess if there were any differences between body sides in individuals. We found no significant differences in mean impedance values between measurement sides, although there was a borderline significant difference between R_∞_ when supine, and R_∞_ and Z_c_ when measured immediately on standing (*p* = 0.10, 0.06, and 0.09, respectively). Nonetheless, limb dominance may not be fully established until 4 to 6 years of age^38^. Furthermore, the consequence of placing BIA electrodes on the left or right side has not been evaluated^3^, although a study in adults found a small difference in impedance between body sides^14^, which is likely related to limb dominance.

This study provides the first evidence to describe the influence of body position on bioimpedance measurements in young children. This study suggests that researchers and clinicians can take bioimpedance measurements without requiring the child to meet various requirements for fasting, voiding, and lying supine for extended periods. Future research is required to confirm these findings and to further evaluate the effect of fasting and voiding on bioimpedance measurements in young children.

## Supplementary Information

Supplementary Information.

## Data Availability

The data will not be openly available as participants did not consent to open access data sharing. Additionally, this is an ongoing longitudinal study in which there will be further future analyses conducted.
