# Supplementary material for: The influence of body position on bioelectrical impedance spectroscopy measurements in young children
Source: Sci Rep. 2021 May 14;11:10346. doi: 10.1038/s41598-021-89568-8 (PMC8121940; doi:10.1038/s41598-021-89568-8)
Supplement: Supplementary file 1 — Supplementary Information. [file 41598_2021_89568_MOESM1_ESM.pdf]

The influence of body position on bioelectrical impedance spectroscopy measurements in young children

Jaz Lyons-Reid[1], Leigh C Ward[1,2], Mya-Thway Tint[3,4], Timothy Kenealy[1,5], Keith M Godfrey[6,7], Shiao-Yng Chan[3,4], Wayne S Cutfield[1,8*]

[1]Liggins Institute, University of Auckland, Auckland, New Zealand

[2]School of Chemistry and Molecular Biosciences, The University of Queensland, Brisbane, Australia

[3]Singapore Institute for Clinical Sciences, Agency for Science, Technology and Research (A*STAR), Singapore

[4]Department of Obstetrics & Gynaecology, Yong Loo Lin School of Medicine, National University of Singapore, Singapore

[5]Department of Medicine and Department of General Practice and Primary Health Care, University of Auckland, Auckland, New Zealand

[6]MRC Lifecourse Epidemiology Unit, University of Southampton, Southampton, United Kingdom

[7]NIHR Southampton Biomedical Research Centre, University of Southampton and University Hospital Southampton NHS Foundation Trust, Southampton, United Kingdom

[8]A Better Start – National Science Challenge, University of Auckland, Auckland, New Zealand

[*]w.cutfield@auckland.ac.nz

Table S1 Bioimpedance body position adjustment equations applied to standing (<1 minute) measurements in validation sub-group (n=15).

	Validation cohort (n = 15)		T-test		Bland-Altman		
	Mean	SD	F	*p*	Bias	Limits of agreement	
						Lower	Upper
R_0							
Supine	798.59	73.21	-1.619	0.128	-5.43<br>-0.68%	-30.87<br>-3.87%	20.02<br>2.51%
Standing (adjusted)	804.01	75.18					
R_∞							
Supine	581.27	62.84	-1.077	0.300	-4.34<br>-0.75%	-34.88<br>-6.00%	26.21<br>4.51%
Standing (adjusted)	585.60	59.56					
Z_c							
Supine	693.22	67.64	-1.441	0.172	-4.75<br>-0.69%	-29.80<br>-4.30%	20.29<br>2.93%
Standing (adjusted)	697.97	67.53					
Z_{50}							
Supine	730.40	71.46	-1.745	0.103	-5.30<br>-0.73%	-28.34<br>-3.88%	17.75<br>2.43%
Standing (adjusted)	735.70	72.61					
R_{50}							
Supine	727.82	71.56	-1.695	0.112	-5.14<br>-0.71%	-28.18<br>-3.87%	17.89<br>2.46%
Standing (adjusted)	732.97	72.61					
Xc_{50}							
Supine	61.01	5.57	0.153	0.881	0.10<br>0.16%	-4.88<br>-8.01%	5.08<br>8.33%
Standing (adjusted)	60.91	4.90					

Abbreviations: R_0, resistance at 0 kHz; R_∞, resistance at infinite kHz; Z_c, impedance at the characteristic frequency; Z_{50}, impedance at 50 kHz; R_{50}, resistance at 50 kHz; Xc_{50}, reactance at 50 kHz.
